# Ceramide induces MMP-9 expression through JAK2/STAT3 pathway in airway epithelium

**DOI:** 10.1186/s12944-020-01373-w

**Published:** 2020-08-24

**Authors:** Lingling Xuan, Feifei Han, Lili Gong, Yali Lv, Zirui Wan, He Liu, Lulu Ren, Song Yang, Wen Zhang, Ting Li, Chunting Tan, Lihong Liu

**Affiliations:** 1 Department of Pharmacy, Beijing Chao-Yang Hospital, Capital Medical University, Beijing, China; 2 Department of Geriatrics, Affiliated Brain Hospital of Nanjing Medical University, Nanjing, China; 3 Department of Respiratory Medicine, Beijing Friendship Hospital, Capital Medical University, Beijing, China

**Keywords:** Ceramide, Airway epithelium, Matrix metalloproteinase 9, Janus tyrosine kinase 2, *Signal transducer and activator of transcription 3*, Airway remodeling

## Abstract

**Background:**

Ceramide, a bioactive lipid, plays an essential role in the development of several pulmonary inflammatory diseases. Matrix metallopeptidase 9 (MMP-9) regulates the synthesis and degradation of extracellular matrix, and is associated with airway remodeling and tissue injury. This study was conducted to investigate the effects and underlying mechanisms of ceramide on MMP-9 expression in airway epithelium.

**Methods:**

BEAS-2B cells, normal human bronchial epithelium cell lines, were pretreated with AG490, a selective janus tyrosine kinase 2 (JAK2) inhibitor, or Stattic, a selective *signal transducer and activator of transcription 3* (STAT3) inhibitor. The cells were then stimulated with C6-ceramide. The levels of MMP-9 were determined by ELISA and real-time quantitative PCR (RT-qPCR). JAK2, phosphorylated JAK2 (p-JAK2), STAT3, and phosphorylated STAT3 (p-STAT3) expression was examined by Western blotting. BALB/c mice were pretreated with AG490 or Stattic before intratracheally instillated with C6-ceramide. Pathological changes in lung tissues were examined by Hematoxylin and Eosin staining, Periodic-acid Schiff staining, and Masson’s trichrome staining. MMP-9, JAK2, p-JAK2, STAT3, and p-STAT3 expression in the lung tissues was examined by Western blotting.

**Results:**

The expression of MMP-9, p-JAK2 and p-STAT3 in BEAS-2B cells was significantly increased after the treatment of C6-ceramide. Furthermore, the increased expression of MMP-9 induced by C6-ceramide was inhibited by AG490 and Stattic. Similar results were obtained in the lung tissues of C6-ceramide-exposed mice which were treated with AG490 or Stattic.

**Conclusions:**

Ceramide could up-regulate MMP-9 expression through the activation of the JAK2/STAT3 pathway in airway epithelium. Targeted modulation of the ceramide signaling pathway may offer a potential therapeutic approach for inhibiting MMP-9 expression. This study points to a potentially novel approach to alleviating airway remodeling in inflammatory airway diseases.

## Introduction

Airway epithelium is a pivotal structure with the greatest surface exposed to a large number of external stimulus, including respiratory viruses, air pollutants, and allergens. The airway epithelium also plays a significant role in the first line of immunological defense by supporting the activation, recruitment, and mobilization of immune cells [1]. However, growing evidence has showed that pulmonary disorders, such as asthma, chronic obstructive pulmonary disease (COPD), and emphysema, are associated with epithelial dysfunction [2–5]. Dysfunctional airway epithelium synthesizes and releases a variety of potent pro-inflammatory mediators such as nitric oxide, chemokine, and cytokines [6, 7]. Moreover, epithelium is believed to participate in airway remodeling through aberrant production of extracellular matrix (ECM), chemokines, growth factors, and matrix metalloproteinases (MMPs) [8, 9].

Ceramide, a bioactive lipid, has been implicated in a variety of physiological and pathological processes, such as cell proliferation, differentiation, apoptosis, and inflammation [10, 11]. Various reports have demonstrated the accumulation of ceramide in prevalent lung diseases, such as asthma, acute lung injury, and COPD [12, 13]. Ceramide is significantly increased in the lungs and serum of patients with asthma and COPD [14, 15]. Ceramide is also associated with the phenotypes of asthma and COPD, and may contribute to exacerbation and poor disease control by worsening airway inflammation [16, 17]. Studies by Oyeniran et al. showed that intratracheal delivery of ceramide in mice promoted pulmonary inflammation and tissue remodeling, and caused airway flow obstruction [18]. In addition, de novo ceramide synthesis contributes to murine lung emphysema development. Ceramide production is markedly increased in the lungs of patients with cigarette smoke-induced emphysema [19]. For the human lung epithelium, an unusual lipid class distribution was found, and ceramide was the predominant sphingolipid [20].

Matrix metalloproteinase 9 (MMP-9), an important enzyme regulating the synthesis and degradation of ECM, is associated with airway remodeling and lung injury in asthma and COPD. MMP-9 directly degrades ECM proteins and is central in facilitating the trafficking of inflammatory cells from the parenchyma to the airways during inflammation [21]. Ceramide metabolism has been linked to MMP-9 expression. Myriocin, a de novo ceramide synthesis inhibitor, showed antioxidant activity and could attenuate MMP-9 expression in cigarette smoke mixture treated human airway epithelium [22]. Buisson-Legendre et al. suggested that ceramide could enhance MMP-9 production in psoriatic keratinocytes [23]. However, the direct effects and mechanisms of ceramide on MMP-9 expression in airway epithelium are not reported.

The objective of this study was therefore to investigate whether ceramide could stimulate MMP-9 expression in airway epithelium. We also aimed to determine the mechanisms of ceramide on MMP-9 expression.

## Materials and methods

### Materials

BEAS-2B cell lines were acquired from the American Type Culture Collection (ATCC, Rockville, MA, USA). C6-ceramide, ammonium pyrrolidinedithiocarbamate (PDTC), tyrphostin B42 (AG490), and 6-Nitrobenzo[b]thiophene-1, 1-dioxide (Stattic) were acquired from Sigma-Aldrich (St Louis, MO, USA). Dulbecco’s Modified Eagle’s Medium (DMEM) and Fetal bovine serum (FBS) were obtained from HyClone (Logan, UT, USA). Anti-JAK2, anti-phosphorylated JAK2 (p-JAK2), anti-STAT3, anti-phosphorylated STAT3 (p-STAT3), anti-CD68, and anti-β-actin antibodies were acquired from Cell Signaling Technology (Beverly, MA, USA). IRDye 680RD Goat anti-Rabbit and Goat anti-Mouse IgG (H + L) was acquired from LI-COR Biosciences (Lincoln, NE, USA). TRIzol was acquired from Invitrogen Corporation (Carlsbad, California, USA). TB Green Mixture was acquired from TaKaRa (Otsu, Shiga, Japan).

### Cell viability assay

Cell viability was determined by the Cell Counting Kit-8 (CCK8) assay. BEAS-2B cells were seeded at a density of 1 × 10^4^ cells/well in 96-well plates and cultured overnight. BEAS-2B cells were then treated with AG490 at 20, 10, 5, or 2.5 μM or Stattic at 2, 1, 0.5, or 0.25 μM for 24 h. We *added* 0.1% DMSO to control wells. The media was then changed to fresh media, and 10 μL of CCK8 solution was added per well. The optical densities (OD) at 450 nm were measured 4 h later using a microplate reader (BioTek, Winooski, VT, USA). Cell viabilities were expressed as percentage of controls cultured with 0.1% DMSO.

### Quantification of MMP-9 in BEAS-2B cells

BEAS-2B cells were seeded at a density of 3 × 10^5^ cells/well in 6-well plates and cultured overnight. BEAS-2B cells were treated with C6-ceramide (10, 5, or 2.5 μM) for 24 h in serum-free media. DMSO (0.1%) was added to control wells. In the experiments involving AG490 and Stattic, AG490 (10, 5, or 2.5 μM) and Stattic (1, 0.5, or 0.25 μM) were added to the cultures 2 h prior to the addition of C6-ceramide. First-strand cDNAs were synthesized using Prime Script RT Reagent kit (Takara Bio, Otsu, Japan). Real-time quantitative PCR (RT-qPCR) was performed using TB Green Mixture (Takara Bio, Otsu, Japan). The primer sequences were as follows: human MMP-9, forward: 5′-GATCATTCCTCAGTGCCGGA-3′, reverse: 5′-TTCAGGGCGAGGACCATAGA-3′; human GAPDH, forward: 5′-CCACATCGCTCAGACACCAT − 3′, reverse: 5′- TTGACGGTGCCATGGAATTT-3′. The relative mRNA levels of MMP-9 were determined with GAPDH as control and were represented as 2^-△△Ct^. MMP-9 protein levels in cell culture supernatant were determined by ELISA (eBioscience, San Diego, CA, USA).

### Quantification of JAK2, p-JAK2, STAT3, and p-STAT3 expression in BEAS-2B

BEAS-2B cells were seeded at a density of 3 × 10^5^ cells/well in 6-well plates and incubated overnight. Then the cells were treated with C6-ceramide (10, 5, or 2.5 μM) for 24 h in serum-free media. DMSO (0.1%) was added to control wells. JAK2, p-JAK2, STAT3, and p-STAT3 levels were examined by Western blotting. Band density was quantified by QuantiScan Version 11 (Biosoft, Cambridge, UK).

### Animal studies

Specifc pathogen free male BALB/c mice (18–20 g, Beijing HFK Bioscience Co, Ltd., Beijing, China) were randomly divided into 4 groups (*n* = 6): the naive normal control group was exposed to vehicle control; the C6-ceramide group was exposed to C6-ceramide and pretreated with 5% DMSO; the AG490 group was exposed to C6-ceramide and was pretreated with AG490 (15 mg/kg i.p., dissolved in 5% DMSO); and the Stattic group was exposed to C6-ceramide and was pretreated with Stattic (5 mg/kg i.p., dissolved in 5% DMSO). These animals were exposed to C6-ceramide at 5 mg / kg of body weight or vehicle control by intratracheal instillation for 3 days. One hour before C6-ceramide exposure on each day, the animals were intraperitoneally injected with AG490, Stattic, or 5% DMSO as indicated above. The lung tissues were then fixed with 4% buffered formalin solution. Pathological changes in the lung tissues were examined using Hematoxylin and Eosin (H&E) staining, Periodic Acid-Schiff (PAS) staining, and Masson’s trichrome staining. CD68 staining was conducted by immunohistochemistry. MMP-9, JAK2, p-JAK2, STAT3, and p-STAT3 levels in lung tissues were examined by Western blotting.

### Statistical analysis

Data were expressed as the mean ± SD or as median with range from three independent experiments. T-test was used to compare means between two groups. One-way ANOVA followed by Tukey post hoc test was used to compare means between three or more groups. Nonparametric data were analyzed using the Kruskal-Wallis test and results were presented as the median and interquartile range. A *P* value of 0.05 or lower was considered statistically significant.

## Results

### C6-ceramide increased MMP-9 expression in BEAS-2B

In order to study the effects of ceramide on MMP-9 expression, human bronchial epithelial BEAS-2B cells were treated with C6-ceramide, a synthetic cell-permeable ceramide analog. The effects of C6-ceramide on MMP-9 expression were determined by RT-qPCR and ELISA, as described in the experimental procedures.

**Fig. 1:**
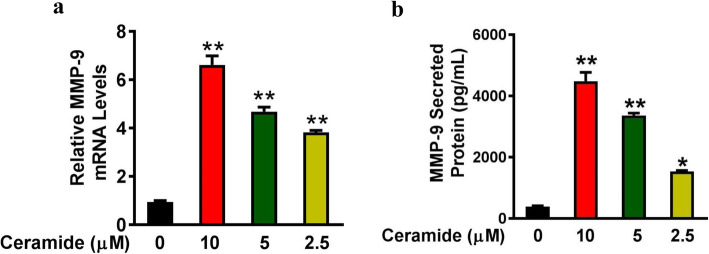
BEAS-2B cells were treated with C6-ceramide (10 μM, 5 μM, or 2.5 μM) or 0.1% DMSO (vehicle) for 24 h. The MMP-9 levels were determined by RT-qPCR (a) and ELISA (b). *n* = 3 per group. ^*^P < 0.05, ^**^P < 0.01 vs controls that were treated with 0.1% DMSO

Compared to cells incubated with DMSO only, treatment with 10 μM, 5 μM, and 2.5 μM of C6-ceramide increased the relative MMP-9 mRNA levels from 1.00 ± 0.09 to 6.62 ± 0.65 (*P* < 0.01), 4.68 ± 0.32 (*P* < 0.01), and 3.82 ± 0.15 (*P* < 0.01), respectively (Fig. 1a), and increased the MMP-9 protein levels from 387.57 ± 40.79 pg/mL to 4481.76 ± 506.40 pg/mL (*P* < 0.01), 3361.10 ± 139.19 pg/mL (*P* < 0.01), and 1536.25 ± 53.05 pg/mL (*P* < 0.01), respectively (Fig. 1b). These data suggested that MMP-9 expression was increased by C6-ceramide in human bronchial epithelial cell line BEAS-2B.

### Inhibition of JAK2 and STAT3 reduced MMP-9 expression in C6-ceramide-treated BEAS-2B

**Fig. 2:**
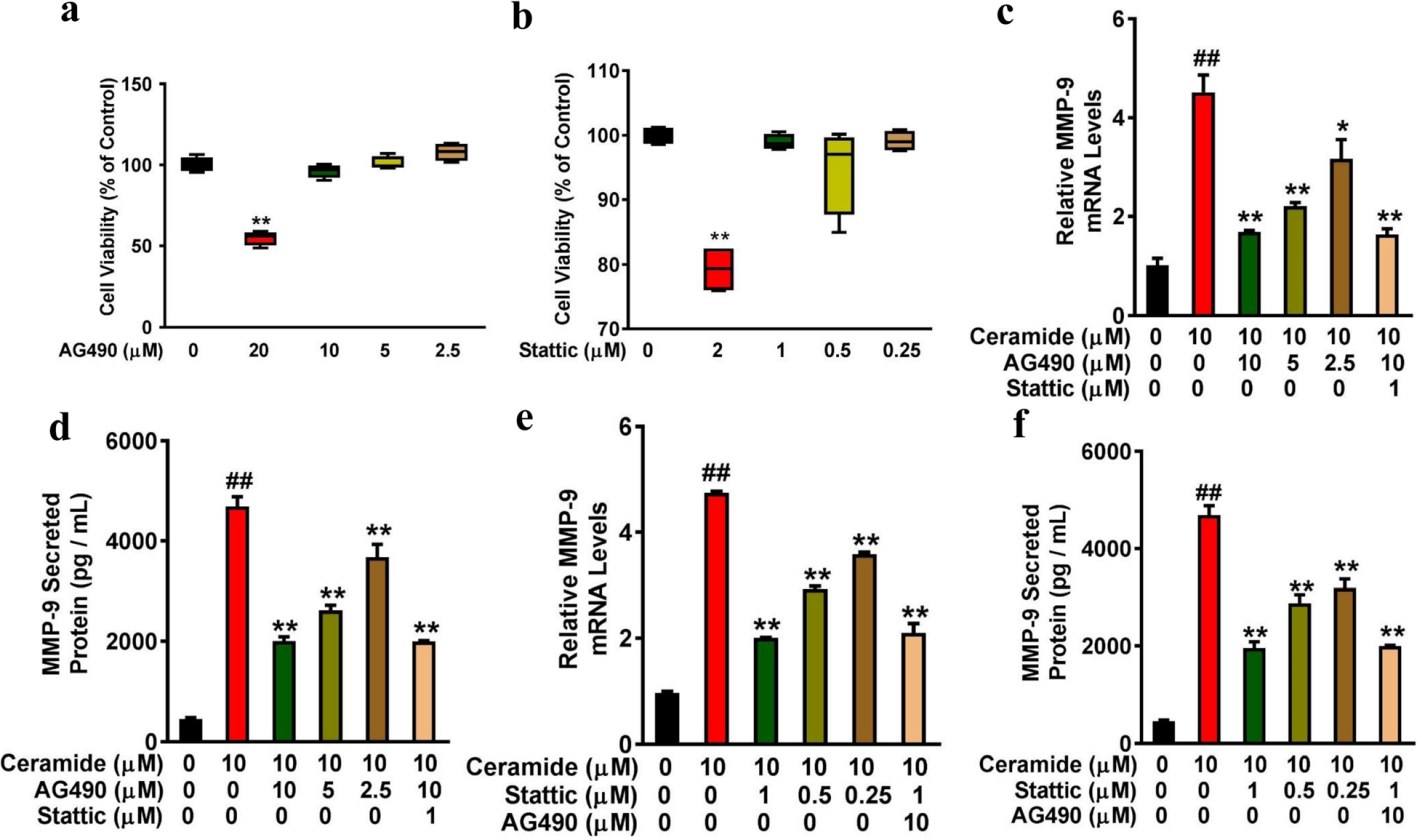
Ceramide-mediated MMP-9 expression was inhibited by JAK2 and STAT3 inhibitors. **(a and b)** BEAS-2B cells seeded on 96-well plates were treated with AG490 (0, 20, 10, 5, or 2.5 μM) **(a)** and Stattic (0, 2, 1, 0.5, or 0.25 μM) **(b)** for 24 h. Cell viability was assessed using the CCK8 assay and expressed as percentage of controls cultured with DMSO. ^**^P < 0.01 vs controls that were cultured with DMSO. **(c and d)** BEAS-2B cells were pretreated with AG490 (0, 10, 5 or 2.5 μM) for 2 h followed by adding 10 μΜ of C6-ceramide for 24 h. MMP-9 levels were determined by RT-qPCR **(c)** and ELISA **(d)**. **(e and f)** BEAS-2B cells were pretreated with Stattic (0, 1, 0.5 or 0.25 μM) for 2 h followed by adding 10 μΜ of C6-ceramide for 24 h. MMP-9 levels were determined by RT-qPCR **(e)** and ELISA **(f)**. n = 3 per group. ^##^*P* < 0.01 vs respective control; ^∗^*P* < 0.05, ^∗∗^*P* < 0.01 vs the C6-ceramide-stimulated group

Ceramide has been reported to induce the activation of various inflammatory signaling pathways, including JAK2/STAT3 and NF-κB [24–26]. Therefore, the involvement of the JAK2/STAT3 and NF-κB pathway in ceramide-induced MMP-9 expression was examined in this study. The cell viability was not significantly affected when BEAS-2B cells were exposed to AG490 (10, 5, or 2.5 μM) and Stattic (1, 0.5, or 0.25 μM) for 24 h (Fig. 2a and b). However, 20 μM of AG490 and 2 μM of Stattic reduced the cell viability of BEAS-2B cells to 55.87% (range 53.74–56.95%) (*P* = 0.04) and 76.10% (range 76.19–82.40%) of control (*P =* 0.01) (Fig. 2a and b), respectively. Therefore, AG490 (10, 5, or 2.5 μM) and Stattic (1, 0.5, or 0.25 μM) were selected for this study.

BEAS-2B cells were pretreated with signal pathway inhibitors, such as PDTC (NF-κB inhibitor), AG490 (JAK2 inhibitor), and Stattic (STAT3 inhibitor), for 2 h before being incubated with 10 μM of C6-ceramide for 24 h. MMP-9 levels were determined by RT-qPCR and ELISA. Compared to the cells incubated with C6-ceramide only, pretreatment with 10 μM, 5 μM, and 2.5 μM of AG490 decreased the relative MMP-9 mRNA levels from 4.52 ± 0.59 to 1.69 ± 0.05 (*P* < 0.01), 2.21 ± 0.12 (*P* < 0.01), and 3.17 ± 0.67 (*P* < 0.01), respectively (Fig. 2c), and decreased the MMP-9 protein levels from 4689.59 ± 335.46 pg/mL to 2010.22 ± 146.44 pg/mL (*P* < 0.01), 2553.77 ± 111.76 pg/mL (*P* < 0.01), and 3674.43 ± 448.99 pg/mL (*P* < 0.01), respectively (Fig. 2d). In addition, combination treatment of BEAS-2B cells with 10 μM of AG490 and 1 μM of Stattic decreased the mRNA and protein level of MMP-9 to 1.64 ± 0.20 (*P* < 0.01) and 1997.43 ± 38.07 pg/mL (*P* < 0.01), respectively, similar to the effect of 10 μM AG490. Meanwhile, when the cells were pretreated with 1 μM, 0.5 μM, and 0.25 μM of Stattic for 2 h, the relative MMP-9 mRNA levels decreased from 4.75 ± 0.05 to 2.00 ± 0.02 (*P* < 0.01), 2.94 ± 0.08 (*P* < 0.01), and 3.59 ± 0.06 (*P* < 0.01), respectively (Fig. 2e), and the MMP-9 protein levels decreased from 4689.59 ± 335.46 pg/mL to 1962.26 ± 223.53 pg/mL (*P* < 0.01), 2879.90 ± 303.29 pg/mL (*P* < 0.01), and 3194.83 ± 318.90 pg/mL (*P* < 0.01), respectively (Fig. 2f). Combination treatment of BEAS-2B cells with Stattic (1 μM) and AG490 (10 μM) decreased the mRNA and protein level of MMP-9 to 2.11 ± 0.31 (*P* < 0.01) and 1997.43 ± 38.07 pg/mL (*P* < 0.01), respectively, similar to the effect of 1 μM Stattic. Meanwhile, AG490 and Stattic did not affect the expression of MMP-9 in the absence C6-ceramide (Fig. S1). Treatment with PDTC, the NF-κB inhibitor, had no significant effect on MMP-9 expression (data not shown). These results suggested that the JAK2/STAT3 signaling pathway may be involved in ceramide-induced MMP-9 expression.

### C6-ceramide induced phosphorylation of JAK2 and STAT3 in BEAS-2B

**Fig. 3:**
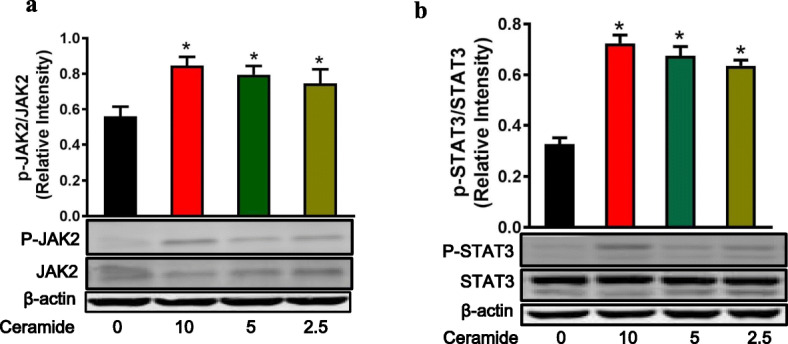
C6-ceramide induces phosphorylation of JAK2 and STAT3 in BEAS-2B cells. After serum starvation for 24 h, BEAS-2B cells were treated with C6-ceramide (10 μM) for 24 h. **(a)** JAK2 and p-JAK2 expression was examined by Western blotting, and relative quantification of the p-JAK2/JAK2 expression was determined by densitometric analysis of the blots. **(b)** STAT3 and p-STAT3 expression was examined by Western blotting, and relative quantification of the p-STAT3/STAT3 expression was determined by densitometric analysis of the blots. n = 3 per group. ^*^P < 0.05 vs controls that were cultured in the absence of C6-ceramide

To confirm these results, the p-JAK2 and p-STAT3 levels were examined. As shown in Fig. 3a, treatment with 10 μM, 5 μM, and 2.5 μM of C6-ceramide increased relative p-JAK2/JAK2 expression from 0.56 ± 0.06 to 0.85 ± 0.05 (*P* = 0.02), 0.79 ± 0.05 (*P* = 0.04), and 0.75 ± 0.08 (*P* = 0.05), respectively (Fig. 3a). In a similar pattern, treatment with 10 μM, 5 μM, and 2.5 μM of C6-ceramide increased p-STAT3/STAT3 expression from 0.33 ± 0.02 to 0.72 ± 0.03 (*P* < 0.01), 0.68 ± 0.04 (*P* < 0.01), and 0.64 ± 0.02 (*P* < 0.01), respectively (Fig. 3b).

### Intratracheal instillation of ceramide increased while AG490 and Stattic administration attenuated MMP-9 expression in vivo

**Fig. 4:**
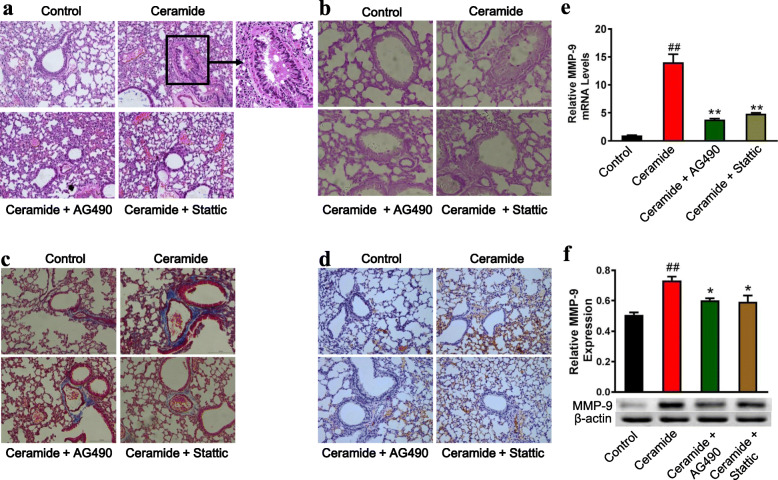
Intratracheal instillation of ceramide increases while AG490 and Stattic administration attenuates MMP-9 expression in vivo. BALB/c mice were treated with AG490 at 15 mg/kg of body weight or Stattic at 5 mg/kg of body weight. The mice were then exposed to 5 mg/kg of C6-ceramide by intratracheal instillation. **(a)** Representative Hematoxylin and Eosin sections of lung tissues (200×). **(b)** PAS staining of lung tissues (200×). **(c)** Masson’s trichrome staining of lung tissues (200×). **(d)** Representative images of CD68 positive cells in the lung tissues. **(e and f)** MMP-9 expression was examined by RT-qPCR **(e)** and Western blotting **(f)**, and quantification of the MMP-9 protein expression was determined by densitometric analysis of the blots. *n* = 6 per group. ^##^*P* < 0.01 vs the control group; ^∗^*P* < 0.05 vs the C6-ceramide-stimulated group

To further investigate the effect and mechanism of ceramide on MMP-9 expression, BALB/c mice were pretreated with AG490 at 15 mg/kg of body weight or Stattic at 5 mg/kg of body weight. BALB/c mice were then exposed to 5 mg/kg of C6-ceramide by intratracheal instillation. C6-ceramide induced a severe inflammatory reaction, characterized by inflammatory cell infiltration, desquamation of bronchial epithelium (Fig. 4a), and mucus production (Fig. 4b). C6-ceramide also induced a dramatic increase in the extent of collagen deposition (Fig. 4c). To visualize inflammatory cell infiltration, the lung tissues were stained with macrophage marker CD68. A highly significant infiltration of CD68 positive cells was observed in C6-ceramide exposed mice lung tissues (Fig. 4d). AG490 at 15 mg/kg of body weight and Stattic at 5 mg/kg of body weight markedly attenuated the C6-ceramide-induced inflammatory cell infiltration, mucus production and collagen deposition in the lung tissues (Fig. 4a-c). The accumulation of CD68 positive cells was also markedly reduced when treated with AG490 and Stattic (Fig. 4d). MMP-9 levels in the lung tissues were examined by RT-qPCR and Western blotting. The MMP-9 mRNA levels significantly increased to 14.03 ± 2.92 in the C6-ceramide-exposed group compared with the vehicle treated mice (*P* < 0.01), while the pretreatment with AG490 and Stattic decreased this level to 3.65 ± 0.28 (*P* = 0.03) and 4.82 ± 0.26 (*P* = 0.02), respectively (Fig. 4e). In a similar pattern, the MMP-9 protein levels significantly increased to 0.73 ± 0.04 in the C6-ceramide-exposed group compared with the vehicle treated mice (0.51 ± 0.03, *P* < 0.01), while the pretreatment with AG490 and Stattic decreased this level to 0.60 ± 0.02 (*P* = 0.03) and 0.59 ± 0.07 (*P* = 0.02), respectively (Fig. 4f).

### Direct intratracheal instillation of ceramide induced phosphorylation of JAK2 and STAT3 in vivo

**Fig. 5:**
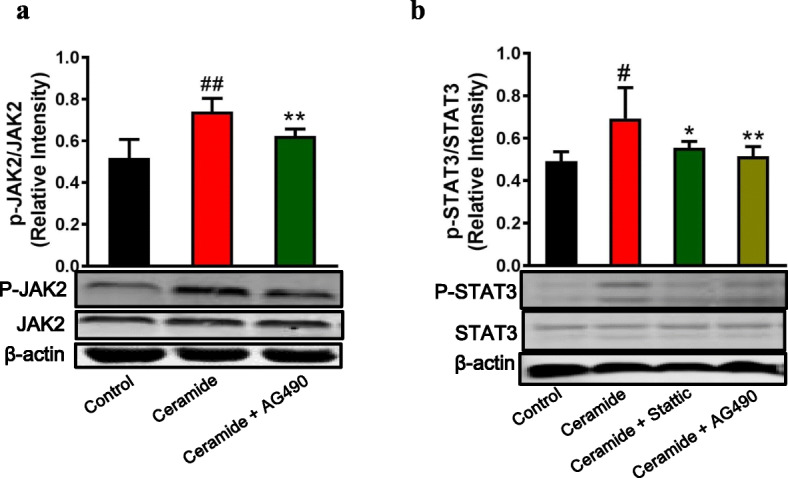
Direct intratracheal instillation of ceramide induces phosphorylation of JAK2 and STAT3 in vivo*.* BALB/c mice were treated with AG490 at 15 mg/kg of body weight or Stattic at 5 mg/kg of body weight. The mice were then exposed to 5 mg/kg of C6-ceramide by intratracheal instillation. **(a)** JAK2 and p-JAK2 expression was examined by Western blotting, and relative quantification of the p-JAK2/JAK2 expression was determined by densitometric analysis of the blots. **(b)** STAT3 and p-STAT3 expression was examined by Western blotting, and relative quantification of the p-STAT3/STAT3 expression was determined by densitometric analysis of the blots. n = 6 per group. ^#^*P* < 0.05, ^##^*P* < 0.01 vs the control group; ^∗^*P* < 0.05, ^**^P < 0.05 vs the C6-ceramide-stimulated group

Consistent with the in vitro studies above, intratracheal instillation of C6-ceramide in BALB/c mice increased relative p-JAK2/JAK2 expression in lung tissues from 0.51 ± 0.08 to 0.74 ± 0.06 (*p* < 0.01) (Fig. 5a), and increased relative p-STAT3/STAT3 expression from 0.49 ± 0.02 to 0.69 ± 0.15 (*p* < 0.01) (Fig. 5b). Treatment with AG490 reduced relative p-JAK2/JAK2 expression in lung tissues from 0.74 ± 0.06 to 0.62 ± 0.03 (*P* = 0.04) (Fig. 5a). In a similar pattern, treatment with AG490 and Stattic reduced relative p-STAT3/STAT3 expression in lung tissues from 0.69 ± 0.15 to 0.51 ± 0.05 (*P* < 0.01) and 0.56 ± 0.03 (*P* = 0.04), respectively (Fig. 5b).

## Discussion

Increased ceramide level is associated with chronic lung diseases, including asthma, COPD, and emphysema [12, 13, 19]. Ceramide has attracted much attention for its potential pro-inflammatory, pro-apoptosis, and pro-oxidant properties [10], while the ceramide-mediated MMP-9 expression in airway epithelium has not been fully studied. This study showed that ceramide promoted MMP-9 production via activation of the JAK2/STAT3 pathway. These data adds ceramide as a novel potent inducer of MMP-9 in the airway epithelium.

MMPs play an essential role in tissue remodeling and they also contribute to excessive breakdown of connective tissue in inflammatory disorders, such as asthma, COPD, and rheumatoid arthritis. MMP-7 and MMP-9 are the predominant forms of MMPs expressed by epithelial cells in lung tissues [27]. MMP-9 degrades elastin, collagen, fibrin, and other extracellular and is central in facilitating the trafficking of inflammatory cells from the parenchyma to the airways during inflammation [21]. Increased level of MMP-9 was found in exhaled breath condensates, bronchoalveolar lavage fluid (BALF), and sputum of patients with asthma and COPD [28]. It is well known that the expression and secretion of MMP-9 in chronic lung diseases is regulated by various stimuli, including IL-1β, TNF-α, and LPSs. Hozumi et al. reported that TNF-α and IL-1β induced MMP-9 expression in human bronchial epithelial cells via NF-κB activation [29]. In addition, Shan et al. demonstrated that platelet activating factor increased MMP-9 expression in human bronchial epithelial cells [30]. Ceramide, a bioactive lipid, has been implicated in a variety of physiological and pathological processes, such as cell proliferation, differentiation, apoptosis, and inflammation. Studies indicate that changes in the ceramide content of the airway epithelium play an essential role in several pulmonary inflammatory diseases. Firstly, ceramide upregulation induces airway epithelium apoptosis. For example, increased ceramide production was observed in the airway epithelium in an experimental asthma model. Inhibition of de novo ceramide synthesis with the ceramide synthase inhibitor fumonisin B1 attenuated oxidative/nitrosative stress, epithelial cell apoptosis, and airway inflammation, and improved the respiratory and histopathological abnormalities [13]. Filosto et al. showed that augmented ceramide production induced the apoptosis of human bronchial epithelial 1 and adenocarcinomic human alveolar basal epithelial cells (A549), and ceramide generation is upstream of the caspase cascades [31]. In addition, our previous studies demonstrated that Vam3, a resveratrol dimer, attenuated ceramide accumulation and inhibited apoptosis in mouse lungs exposed to cigarette smoke [32]. Secondly, increased ceramide generation triggers oxidative stress. Ceramide was reported to induce oxidative damage by increasing ROS generation or by decreasing ROS elimination [33]. Previous studies suggested that ceramide promoted oxidative stress via generation of superoxide, and contributed to cigarette smoke-induced lung injury [34]. Thirdly, increased ceramide concentration in airway epithelium triggered chronic pulmonary inflammation [35]. These studies indicated that the increased production of ceramides in the airway epithelium played an important role in apoptosis, oxidative stress and inflammation. However, the ceramide-mediated MMP-9 expression in airway epithelium has not been fully studied. In this study, the mRNA levels of MMP-9 and MMP-7 were determined in BEAS-2B cells using RT-qPCR. Ceramide did not affect the mRNA levels of MMP-7 (data not shown). However, ceramide was a novel potent inducer of MMP-9 in airway epithelium. C6-ceramide significantly increased MMP-9 expression in BEAS-2B cells. Direct intratracheal instillation of ceramide induced MMP-9 expression in mouse lungs, and promoted inflammatory cells infiltration, mucus production and collagen deposition.

Ceramide could induce the activation of various inflammatory signaling pathways, including NLRP3/Caspase1/IL-1β, JAK/STAT and NF-κB [24–26, 36]. Grassmé et al. showed the activation of the NLRP3 inflammasome by the increased ceramide levels in Cystic Fibrosis airways [37]. The ceramide-induced activation of NF-κB promotes the production of several inflammatory mediators and adhesion molecules [26]. Kim et al. reported that ceramide up-regulated MMP-1 expression via activation of JAK1/STAT-1 pathway in human dermal fibroblasts [25], while Reunanen et al. reported that enhancement of MMP-1 gene expression by ceramide was mediated via extracellular signal-regulated and stress-activated protein kinase pathways [38]. In preliminary experiment, C6-ceramide treatment activated phosphorylation of JAK1 and JAK2 in airway epithelium. Meanwhile, C6-ceramide stimulated the phosphorylation of JAK2 to a significantly larger extent than JAK1 in BEAS-2B cells (data not shown). C6-ceramide-induced MMP-9 expression was significantly decreased by JAK2 inhibitor AG490 but not by NF-κB inhibitor PDTC. Once activated, JAK2 initiates a number of downstream molecules, including STAT3 and STAT5. STAT3 is a key player in epithelial cell migration and is required for repair of the bronchiolar epithelium [39]. Moreover, activation of STAT3 adjusts and controls MMP-9 expression [27, 40]. This study showed that p-STAT3 levels were remarkably elevated in ceramide treated BEAS-2B cells and mice lungs. Static, the selective STAT3 inhibitor, was used to verify the involvement of STAT3 in MMP-9 expression. The results demonstrated that C6-ceramide-induced MMP-9 expression was significantly decreased by Stattic. Consistent with the in vitro studies above, direct intratracheal instillation of ceramide induced a significant increase in MMP-9 expression in the lungs, and caused significant pulmonary inflammation, mucus hypersecretion, and collagen deposition which can be seen in the histopathological data. Administration of AG490 and Stattic reduced the levels of p-JAK2 and p-STAT3, and ameliorated MMP-9 expression and airway remodeling. Meanwhile, AG490 and Stattic ameliorated ceramide-induced pathologic changes, including mucus hypersecretion and collagen deposition. JAK2/STAT3 is an important signaling pathway to regulate multiple gene expression, including MMP-9. This study demonstrated that C6-ceramide treatment significantly activated the JAK2/STAT3 signaling. C6-ceramide mediated MMP-9 expression was inhibited by JAK2/STST3 signaling inhibitors (AG490 and Stattic). However, C6-ceramide did not affect the expression of MMP-9 in the absence C6-ceramide.

### Study strengths and limitations

The strengths of this study were listed as following: First, the study reported the effect of ceramide on MMP-9 production in airway epithelium which has not been reported. Second, this study clarify the pathway involved in ceramide-induced MMP-9 expression in airway epithelium. Some limitations of the study deserve to be mentioned. Firstly, JAK2 knock-out mice and STAT3 knock-out mice are needed to confirm these results. Secondly, MMP-9 expression was only partly inhibited by AG490 and Stattic. In airway epithelium, other signaling pathway may be involved in ceramide-induced MMP-9 expresison. Thus, further studies are needed to investigate the involvement of other signaling pathway in ceramide-induced MMP-9 expression in airway epithelium.

## Conclusions

In conclusion, the findings of the present study suggest that ceramide may induce MMP-9 expression in airway epithelium via the JAK2/STAT3 pathway. Therefore, targeted modulation of the ceramide signaling pathway may offer a potential therapeutic approach for inhibiting MMP-9 expression in airway epithelium. Further understanding of the mechanisms responsible for pathological remodeling may highlight novel therapeutic strategies in chronic inflammatory airway disease, such as asthma and COPD.

## Supplementary information

**Additional file 1: Figure S1.**

## Data Availability

Data are available from the authors on request.

## Abbreviations

BALF: Bronchoalveolar Lavage Fluid
COPD: Chronic Obstructive Pulmonary Disease
DMEM: Dulbecco’s Modified Eagle’s Medium
ECM: Extracellular Matrix
FBS: Fetal Bovine Serum
JAK2: Janus Tyrosine Kinase 2
MMPs: Matrix Metalloproteinases
MMP-9: Matrix Metallopeptidase 9
RT-qPCR: Real-Time Quantitative PCR
STAT3: Signal Transducer and Activator of Transcription 3
