# Supplementary figures and images for: Ceramide induces MMP-9 expression through JAK2/STAT3 pathway in airway epithelium
Source: Lipids Health Dis. 2020 Aug 24;19:196. doi: 10.1186/s12944-020-01373-w (PMC7444274; doi:10.1186/s12944-020-01373-w)

## Slide 1
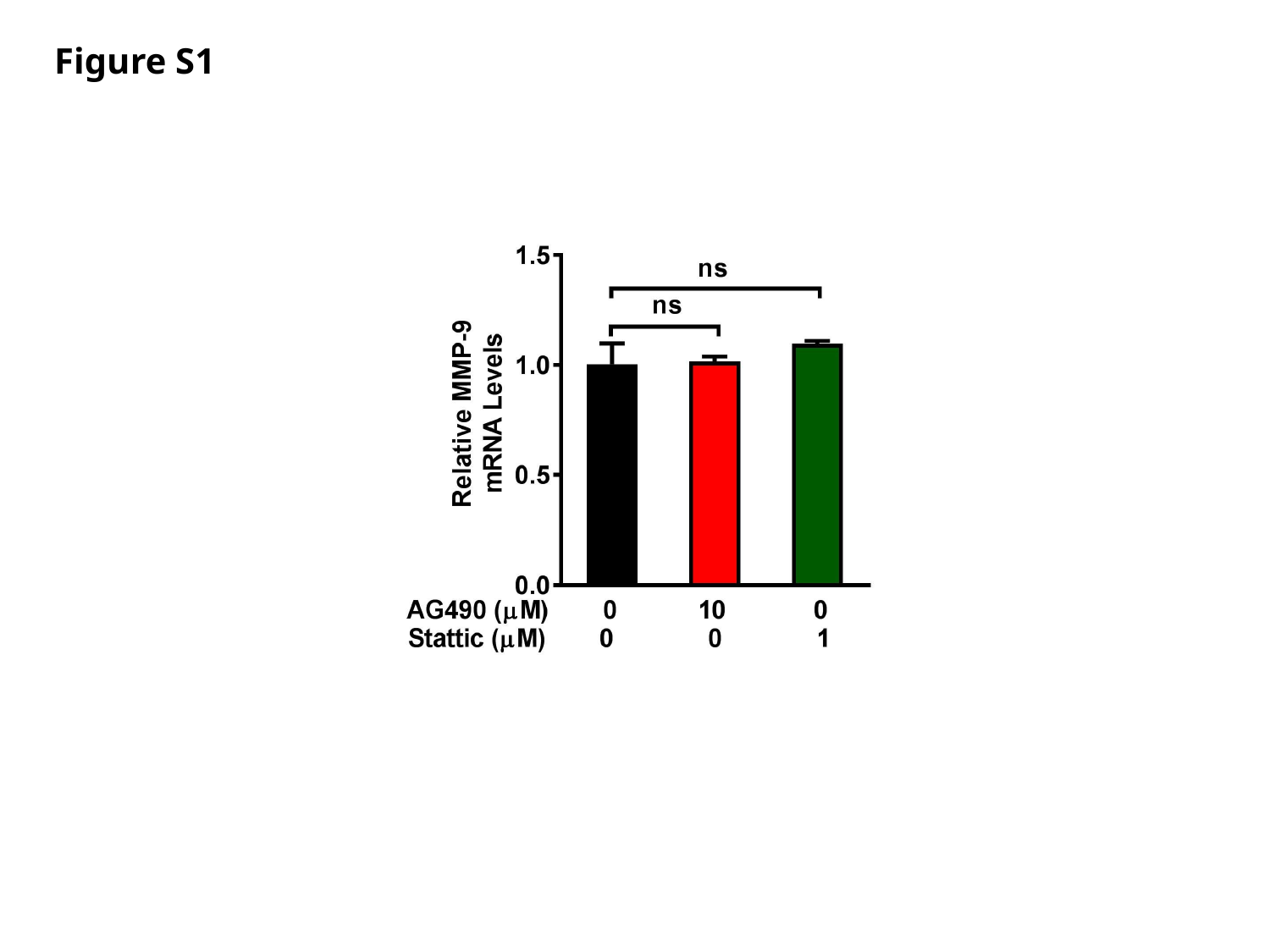

Figure S1

Supplement: Supplementary file 1 — Additional file 1: Figure S1. [file 12944_2020_1373_MOESM1_ESM.pptx]
